# Supplementary material for: Post‐exercise hot water immersion enhances haemodynamic and vascular benefits of exercise without further improving cardiorespiratory fitness, glucose, lipids or inflammation
Source: J Physiol. 2025 Jul 28;603(16):4515–33. doi: 10.1113/JP288873 (PMC12369307; doi:10.1113/JP288873)
Supplement: Supplementary file 2 — Table S1. Changes in the 36‐Item Short Form Health Survey domains following 8 weeks of post‐exercise hot water immersion (EX+HWI) and post‐exercise thermoneutral water immersion (EX+TWI). Table S2. Changes in basic physiological and perceptual measures following 8 weeks of post‐exercise hot water immersion (EX+HWI) and post‐exercise thermoneutral water immersion (EX+TWI). [file TJP-603-4515-s002.pdf]

Supplementary data

Table 1. Changes in the 36-Item Short Form Health Survey domains following 8-weeks of post-exercise hot water immersion (EX+HWI) and post-exercise thermoneutral water immersion (EX+TWI).

	**EX+HWI**			**EX+TWI**			**Statistical significance**
	Week 0	Week 8	Pre to post median difference and Q1-Q3	Week 0	Week 8	Pre to post median difference and Q1-Q3	Delta change between-groups
**SF-36 Health Survey**							
Physical health component (a.u.)	75 ± 14	82 ± 9	7 [Q1: 2, Q3: 17]	79 ± 8	80 ± 8	1 [Q1: 0, Q3: 3]	***P* = 0.036**
Mental health component (a.u.)	72 ± 11	82 ± 6	10 [Q1: 5, Q3: 15]	75 ± 11	80 ± 7	5 [Q1: 1, Q3: 7]	*P* = 0.056
Physical functioning (a.u.)	85 ± 15	95 ± 15	10 [Q1: 0, Q3: 13]	90 ± 13	90 ± 8	0 [Q1: 0, Q3: 5]	*P* = 0.628
Physical role limitations (a.u.)	100 ± 6	88 ± 28	–12 [Q1: –28, Q3: 0]	94 ± 12	94 ± 6	0 [Q1: 0, Q3: 6]	*P* = 0.908
Bodily pain (a.u.)	80 ± 44	90 ± 16	10 [Q1: 0, Q3: 27]	90 ± 16	90 ± 10	0 [Q1: 0, Q3: 10]	*P* = 0.368
Emotional role limitations (a.u.)	100 ± 25	100 ± 10	0 [Q1: 0, Q3: 13]	100 ± 17	100 ± 10	0 [Q1: 0, Q3: 9]	*P* = 0.716
Vitality (a.u.)	69 ± 16	78 ± 9	9 [Q1: 3, Q3: 22]	69 ± 25	72 ± 9	3 [Q1: –3, Q3: 9]	*P* = 0.171
Social functioning (a.u.)	88 ± 25	100 ± 0	12 [Q1: 0, Q3: 19]	100 ± 12	100 ± 12	0 [Q1: 0, Q3: 6]	*P* = 0.157
Mental health (a.u.)	85 ± 15	88 ± 15	3 [Q1: 0, Q3: 10]	85 ± 13	90 ± 5	5 [Q1: 3, Q3: 10]	*P* = 0.917
General health (a.u.)	45 ± 8	54 ± 10	9 [Q1: 3, Q3: 10]	45 ± 5	50 ± 5	5 [Q1: 0, Q3: 5]	*P* = 0.160

The 36-Item Short Form Health Survey domains in the EX+HWI (n = 11; 5 females) and EX+TWI (n = 11; 6 females) groups. Data are expressed as medians ± IQR. *P*-values represent between-group differences using Quade's ANCOVA on delta change scores from pre to post intervention, with group as the fixed factor, and baseline data as the covariate.

Table 2. Changes in basic physiological and perceptual measures following 8-weeks of post-exercise hot water immersion (EX+HWI) and post-exercise thermoneutral water immersion (EX+TWI).

	**EX+HWI**						**EX+TWI**						**Statistical significance**	
	**Week 0**			**Week 8**			**Week 0**			**Week 8**				
	Pre	Post	Pre to post mean/median difference and 95% CIs/Q1-Q3	Pre	Post	Pre to post mean/median difference and 95% CIs/Q1-Q3	Pre	Post	Pre to post mean/median difference and 95% CIs/Q1-Q3	Pre	Post	Pre to post mean/median difference and 95% CIs/Q1-Q3	Week 0 delta change between-groups	Week 8 delta change between-groups
Tympanic temperature (°C)	36.6 ± 0.4	37.6 ± 0.6	1 [0.7, 1.3]	36.5 ± 0.4	38.3 ± 0.5	1.8 [1.3, 2.1]	36.6 ± 0.4	36.8 ± 0.3	0.2 [0, 0.4]	36.4 ± 0.2	36.6 ± 0.3	0.2 [0, 0.4]	***P* < 0.001**	***P* < 0.001**
Heart rate (bpm)	76 ± 12	98 ± 13	22 [15, 30]	71 ± 7	105 ± 11	34 [24, 42]	74 ± 8	78 ± 6	2 [–5, 6]	74 ±6	75 ±12	1 [–7, 8]	***P* < 0.001**	***P* < 0.001**
Thermal sensation (a.u.)	0 ± 1	3 ± 1	3 [Q1: 3, Q3: 4]	0 ± 1	4 ± 1	4 [Q1: 3, Q3: 5]	0 ± 1	0 ± 1	0 [Q1: –1, Q3: 2]	1 ± 1	1 ± 1	0 [Q1: –1, Q3: 0]	***P* < 0.001**	***P* < 0.001**
Thermal comfort (a.u.)	1 ± 2	3 ± 2	2 [Q1: –1, Q3: 3]	2 ± 2	–1 ± 3	–3 [Q1: –3, Q3: –1]	1 ± 2	1 ± 2	0 [Q1: –1, Q3: 1]	1 ± 1	2 ± 2	1 [Q1: 0, Q3: 2]	*P* = 0.093	***P* = 0.002**
Basic affect (a.u.)	3 ± 1	3 ± 1	0 [Q1: 0, Q3: 1]	3 ± 2	2 ± 2	–1 [Q1: –2, Q3: 0]	3 ± 1	4 ± 1	1 [Q1: 0, Q3: 1]	3 ± 2	3 ± 2	1 [Q1: –1, Q3: 1]	*P* = 0.629	*P* = 0.527
Physical Activity Enjoyment Scale (a.u.)	n/a	42 ± 10	[Q1: 37, Q3: 47]	n/a	42 ± 14	[Q1: 37, Q3: 54]	n/a	43 ± 8	[Q1: 36, Q3: 49]	n/a	39 ± 15	[Q1: 29, Q3: 52]	*P* = 0.522	*P* = 0.695

Basic physiological and perceptual measures in the EX+HWI (n = 12; 6 females) and EX+TWI (n = 12; 7 females) groups. Data are expressed as means ± SD or medians ± IQR. *P*-values represent between-group differences using Paired t-tests or Mann-Whitney U tests.
